# Exploratory Agreement of 3D Body Surface Analysis with Expert Manual Assessment of Pelvic Malalignment in Patients with Non-Acute Low Back Pain: A Prospective Pilot Study with Linked Post-Hoc Analyses

**DOI:** 10.3390/diagnostics16203293

**Published:** 2026-10-10

**Authors:** Yeon-Woo Lee, Yeong-Jae Shin, Bong-Jin Shin, Sun-Young Park, In-Hyuk Ha, Jun-Su Jang, Sang-Hun Lee, Seong-Su Joo, In Heo, Man-Suk Hwang, Byung-Cheul Shin

**Affiliations:** 1 School of Korean Medicine, Pusan National University, Yangsan 50612, Republic of Korea; lyw2667@gmail.com (Y.-W.L.); shl0305@gmail.com (S.-Y.P.); drheoin@pusan.ac.kr (I.H.); 2 Department of Korean Medicine Rehabilitation, Spine and Joint Center, Pusan National University Korean Medicine Hospital, Yangsan 50612, Republic of Korea; shinyj5107@gmail.com (Y.-J.S.); shinbj0831@gmail.com (B.-J.S.); 3 Jaseng Spine and Joint Research Institute, Jaseng Medical Foundation, Seoul 06110, Republic of Korea; hanihata@jaseng.org; 4 Digital Health Research Division, Korea Institute of Oriental Medicine, Daejeon 34054, Republic of Korea; junsu.jang@kiom.re.kr; 5 Korean Medicine Data Division, Korea Institute of Oriental Medicine, Daejeon 34054, Republic of Korea; ezhani@kiom.re.kr; 6 Team Elysium Inc., Seoul 06682, Republic of Korea; seongsu.joo@teamelysium.kr

**Keywords:** 3D body surface analysis, Chuna manual therapy, non-acute low back pain, pelvic malalignment, X-ray

## Abstract

**Background/Objectives:** We aimed to explore whether selected non-invasive three-dimensional (3D) body surface indicators differed between prospectively defined enrollment groups and, in linked post-hoc analyses, to identify candidate indicators associated with expert manual judgment. **Methods:** At screening (visit 1), 88 participants underwent manual examination and pelvic X-ray assessment. Of these, 60 entered the prospective pilot study and underwent 3D body surface analysis at visit 2. The prespecified prospective comparison used the protocol-defined malalignment group (*n* = 30) and comparison group (*n* = 30). Final manual diagnosis served as an expert-judgment reference comparator. For post-hoc exploratory ROC analyses, the same 60 participants were reclassified according to final manual diagnosis alone as manual-positive (*n* = 40) or manual-negative (*n* = 20). **Results:** In the prespecified prospective comparison, neither 3D surface-height asymmetry nor marker-angle outcomes differed significantly between the protocol-defined enrollment groups across postures P1–P4. Among all 88 screened participants, X-ray sensitivity and specificity relative to final manual diagnosis were 88.6% and 34.1%, respectively. In the post-hoc exploratory ROC analyses, IC surface-height difference at P3 showed an apparent AUC of 0.684 (*p* = 0.015), and the highest individual marker-angle AUC was 0.706 at P2 (*p* = 0.005). An exploratory five-variable model, developed and evaluated in the same dataset, yielded an apparent in-sample AUC of 0.756. **Conclusions:** The prespecified 3D comparisons did not distinguish the protocol-defined enrollment groups. Post-hoc analyses of the same 60 participants identified candidate 3D indicators associated with expert manual judgment, but these findings were exploratory and do not establish diagnostic accuracy or clinical utility. Prospective validation using prespecified indicators and thresholds, an appropriate independent or composite reference standard, and formal reliability assessment is required before a clinical role for 3D body surface analysis can be determined.

## 1. Introduction

Pelvic malalignment—including innominate rotation—is considered a potential contributing or aggravating factor for low back pain (LBP), and Chuna manual therapy (CMT), a key Korean medicine intervention, aims to alleviate LBP by correcting this malalignment [1,2]. Since its inclusion in the National Health Insurance system in 2019 [3], demand for standardized diagnostic criteria has increased [4]. Previous research has also highlighted the need for more standardized approaches to pelvic malalignment diagnosis [5]. Current clinical practice relies on palpation-based methods, such as standing/sitting flexion tests, prone leg length assessments, and long-sitting tests [6]. However, no objective gold standard exists for diagnosing non-fracture pelvic malalignment, representing a critical clinical gap [4,7]. Although manual assessment is widely used, it has low reproducibility and is highly examiner-dependent [7,8,9,10,11]; however, its accessibility supports its continued use as a pragmatic component of clinical assessment.

X-ray imaging is the primary method for assessing pelvic malalignment, including innominate anterior/posterior rotation and in-flare/out-flare displacement [12,13,14]. However, its use is limited to static, single time-point evaluation owing to radiation exposure concerns and logistical constraints [15]. Notably, X-rays do not capture dynamic functional features, such as soft tissue asymmetry or postural variation, which are central to CMT. This difference between structural imaging and functional clinical assessment may complicate interpretation when the two modalities yield discordant findings.

Three-dimensional (3D) body surface analysis is a non-invasive approach for characterizing surface posture and alignment-related features [16]. Prior research has been largely focused on macroscopic applications (e.g., scoliosis and gait) [17,18]. Earlier approaches relying on simplistic surface height metrics showed susceptibility to soft-tissue artifacts [16].

Therefore, 3D analysis may offer a complementary, non-radiographic approach to characterizing pelvic alignment, pending further validation. It may also hold potential for future quantitative longitudinal monitoring of alignment changes during CMT, although this monitoring application was not directly evaluated in the present study and would require dedicated repeatability and responsiveness studies. A previous study using a body-shape analysis system reported significant correlations with X-ray measurements for pelvic obliquity, ASIS–PSIS angle, and lumbar lateral flexion angle [19].

Because multimodal comparisons integrating 3D surface metrics with radiographic and manual assessments remain scarce [4], this study evaluated surface-height measures and angle-based measures derived from the relative positions of the posterior superior iliac spine (PSIS) and iliac crest (IC) landmarks across four standardized postures (P1–P4). The prespecified prospective analysis examined whether these 3D indicators differed between the protocol-defined malalignment and comparison groups. In linked post-hoc exploratory analyses, the same participants were reclassified according to final manual diagnosis to identify candidate 3D indicators associated with expert manual judgment for future prospective validation.

## 2. Materials and Methods

### 2.1. Study Design and Participants

This study comprised a prospective pilot component and linked exploratory analyses. At screening (visit 1), 88 participants underwent manual examination and pelvic X-ray assessment. Screening data from all 88 participants were subsequently used to evaluate manual diagnostic reliability and the agreement of X-ray assessment with final manual diagnosis.

Of the 88 screened participants, 60 were enrolled in the prospective pilot study according to the protocol-specified enrollment algorithm and completed 3D body surface analysis at visit 2. Thirty participants were assigned to the protocol-defined malalignment group and 30 to the comparison group. The malalignment group consisted of participants meeting the protocol-defined positive classification. According to the screening classification records, the comparison group comprised 9 participants in the definite-negative category and 21 participants allocated to the protocol-defined subgroup-analysis category. This protocol-based classification was used exclusively for the prespecified prospective comparison.

Recruitment occurred at Pusan National University Korean Medicine Hospital from 5 September 2022, to 6 April 2023 (*n* = 37 screened; *n* = 25 enrolled) and at Jaseng Hospital of Korean Medicine from 13 October 2022, to 21 June 2023 (*n* = 51 screened; *n* = 35 enrolled). Participants were recruited competitively across the two participating institutions. The remaining 28 screened participants (*n* = 12 at Pusan and *n* = 16 at Jaseng) were not enrolled in the prospective pilot study because the recruitment quota for the comparison group had been reached at both institutions. These participants contributed to the manual diagnostic reliability and X-ray agreement analyses but did not undergo 3D body surface analysis.

No formal a priori sample-size calculation based on a specified effect size, alpha level, and statistical power was performed because no comparable prior study was available for the primary surface-height outcome. Considering the study period, available resources, and recruitment feasibility, an initial target of 25 participants per group (total *n* = 50) was selected for this pilot study. After allowing for an anticipated dropout rate of approximately 15%, the enrollment target was increased to 30 participants per group (total *n* = 60). No enrolled participant dropped out, and all 60 participants completed visit 2. This sample size was intended to support the prespecified inter-group comparison of 3D surface indicators and to generate preliminary effect-size estimates for planning a future definitive study, rather than to provide confirmatory power for ROC-based or diagnostic-performance analyses.

The prespecified prospective analysis compared the protocol-defined malalignment and comparison groups (30 participants per group). Separately, for the post-hoc exploratory 3D ROC analyses, the same 60 participants were reclassified according to final manual diagnosis alone, yielding 40 manual-positive and 20 manual-negative participants.

This prospective trial was approved by the Institutional Review Boards (IRBs) of Pusan National University Korean Medicine Hospital and Jaseng Hospital of Korean Medicine (IRB No. PNUKHIRB 2022-04-001, approved 19 May 2022; JASENG 2022-04-011, approved 20 May 2022) and registered with the Clinical Research Information Service (CRIS; Registration No. KCT0007480). Secondary analysis of the screening data received an IRB exemption from Pusan National University Korean Medicine Hospital (IRB No. PNUKHIRB 2025-12-005, approved 5 January 2026), as it involved the secondary use of previously collected clinical trial data, and was registered with CRIS (Registration No. KCT0012065). All procedures followed the principles of the Declaration of Helsinki. Written informed consent was obtained from all participants before enrollment. Baseline demographic and clinical characteristics of the 60 participants are presented in Supplementary Table S1, and the STARD 2015 checklist is provided in Supplementary Table S2.

### 2.2. Inclusion and Exclusion Criteria

The inclusion criteria were as follows: (1) age 19–65 years; (2) non-acute LBP lasting more than 2 weeks after symptom onset, with a numeric rating scale (NRS) score of ≥4; (3) a body mass index (BMI) of <30 kg/m^2^; (4) ability to communicate normally; and (5) provision of voluntary written informed consent. The cutoff of 30 kg/m^2^ corresponds to the lower boundary of class 2 obesity in the Korean Society for the Study of Obesity clinical practice guideline [20]. This study-specific eligibility threshold was intended to reduce potential surface-measurement variability associated with excess subcutaneous soft tissue and to facilitate palpation of deep landmarks, including the PSIS and IC.

The exclusion criteria were as follows: (1) an underlying condition that could account for LBP other than pelvic malalignment, including spinal metastasis, acute fracture, spinal dislocation, or poliomyelitis; (2) absolute contraindications to CMT, including acute cauda equina syndrome, spinal dislocation, or cerebral aneurysm; (3) a history of lumbosacral, pelvic, or knee surgery; congenital spinal disease; or nerve root block procedures; (4) planned surgery or procedures during the study period; (5) chronic conditions likely to affect study outcomes, including major cardiovascular or renal disease, diabetic neuropathy, dementia, or epilepsy; (6) CMT treatment for LBP within 2 weeks before enrollment; (7) participation in another interventional clinical trial within 1 month before enrollment; (8) inability to communicate normally because of dementia, mild cognitive impairment, or another condition; (9) pregnancy; or (10) inability to provide informed consent or any other condition considered by the investigators to preclude appropriate study conduct.

### 2.3. Study Procedures

At screening (visit 1), all participants underwent standing anteroposterior (AP) pelvic X-ray imaging. Pelvic X-rays were interpreted by one designated reader at each institution, with different readers across the two participating sites. The designated readers did not participate in the manual examinations or 3D body surface measurements and applied a shared standard operating procedure (SOP) for pelvic X-ray assessment. During the study period, one designated reader was replaced following completion of residency training. The incoming reader applied the same shared SOP. X-ray interpretations were performed independently and without access to the manual examination or 3D measurement results.

Manual examinations were conducted independently by two Korean Medicine doctors according to the same SOP, with blinding to each other’s assessments and to X-ray findings. Intra-rater reliability was assessed using repeated manual examinations, and inter-rater agreement was assessed between the two clinicians.

Surface markers were placed on the skin in the pelvic region according to a common, explicitly defined palpation-based guideline applied identically at both sites: (a) PSIS1 and PSIS2 were identified by palpation; (b) L4 was designated as the spinous process palpable approximately 6–7 cm superior to the midpoint of the line connecting the bilateral PSIS landmarks; (c) L1 was identified by counting the third spinous process superior to L4; and (d) IC1 and IC2 were designated at bilaterally symmetric distances of 7 cm from L4. Circles with a diameter of 12 mm were drawn directly on the skin using eyeliner at each marked landmark.

**Figure 1:**
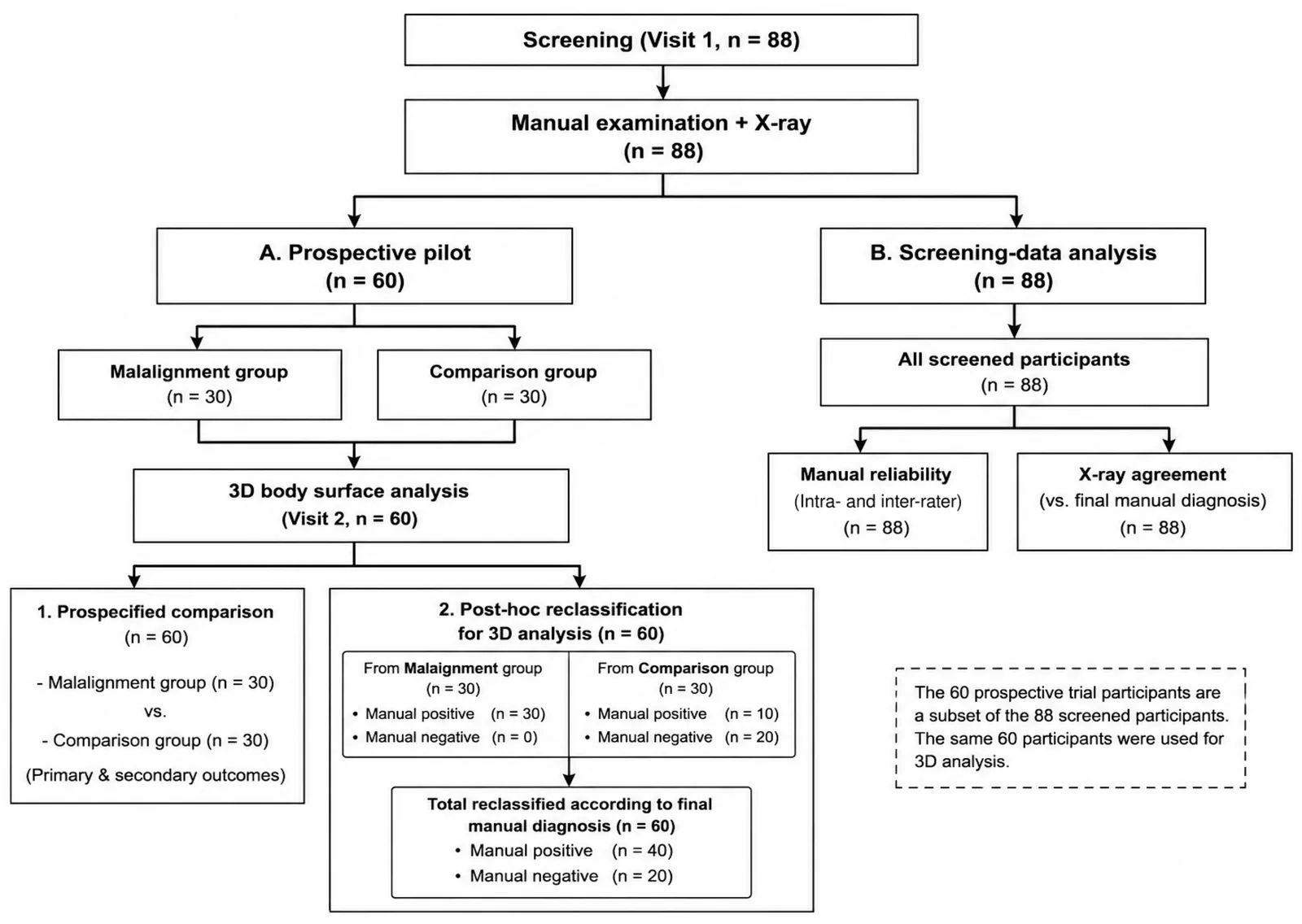
Flowchart of study participants and data analysis.

Each participant underwent one 3D acquisition in each of the four standardized postures (P1–P4). No repeated measurements were performed for averaging or reliability assessment, and no scan required reacquisition after quality verification. All 3D scans were performed using identical equipment and settings. Point-cloud data for the PSIS and IC regions were extracted using an automated algorithm. A flowchart of the study is shown in Figure 1.

### 2.4. Diagnostic Methods for Pelvic Malalignment

We utilized three modalities to evaluate pelvic malalignment: radiographic (X-ray) analysis, manual examination by Korean Medicine doctors, and 3D body surface analysis.

#### 2.4.1. Manual Examination

Manual examinations were performed according to a standard operating procedure (SOP) developed through expert consensus based on prior research. For the purposes of this study, pelvic malalignment was operationally defined as a clinically assessed pattern of asymmetric pelvic landmark movement and relative positional findings involving innominate anterior/posterior rotation and in-flare/out-flare deviation, as identified using the standardized manual examination framework described below. This operational definition should not be interpreted as an independently validated structural diagnosis.

The assessment followed a structured three-step protocol: (1) pelvic malalignment, (2) innominate anterior/posterior rotation, and (3) in-flare/out-flare deviation.

(1) Assessment of Pelvic Malalignment: The primary assessment—the standing flexion test—was conducted according to established sacroiliac joint assessment procedures [6,21]. Malalignment was determined visually and tactilely by observing the relative superior movement of one PSIS compared with the contralateral side during trunk flexion [21]. To ensure objectivity, skilled clinicians established a clinical threshold for “distinct movement” through prior training. A positive result was defined as clear upward PSIS excursion during the terminal phase of flexion. In ambiguous cases, the sitting flexion test was used as a supplementary assessment to identify sacroiliac joint dysfunction associated with hypomobility and determine the affected side, with the specific rotational pattern confirmed in the subsequent step. All manual examinations were performed independently by two Korean Medicine doctors under blinded conditions, and their assessments were compared to evaluate inter-rater agreement. In the exploratory analyses, the manual diagnosis used for analysis was treated as an expert-judgment reference comparator for the X-ray and 3D analyses, rather than as an independently validated diagnostic gold standard.
(2) Innominate Anterior/Posterior Rotation: The primary assessment was the long-sitting test: a leg that appeared shorter in the supine position but lengthened upon sitting was interpreted as indicating posterior rotation, whereas the opposite pattern was interpreted as indicating anterior rotation [21]. When the primary assessment was inconclusive, two supplementary assessments were used: (i) the relative height difference between the ASIS and the PSIS, and (ii) a prone functional leg-length analysis [14], in which bilateral leg length was compared with the knees fully extended and then flexed to 90° in the prone position. Lengthening (or persistent shortening) of the initially shorter leg after knee flexion was interpreted as suggesting posterior rotation; equal leg length in extension that became unequal after knee flexion was interpreted as suggesting anterior rotation.
(3) In-flare/Out-flare Assessment: Flare deviation was assessed using the hip abduction external rotation test, which measures the angle of leg abduction with a firm ligamentous end feel in the prone position. To ensure reproducibility, clinicians calibrated the tactile criteria for identifying resistance points. According to established criteria, an abduction angle ≥40° was defined as in-flare, ≤30° as out-flare, and 30–40° was considered neutral [2]. In cases of ambiguity, the umbilicus–ASIS distance was measured to aid clinical judgment.

#### 2.4.2. X-Ray Assessment

**Figure 2:**
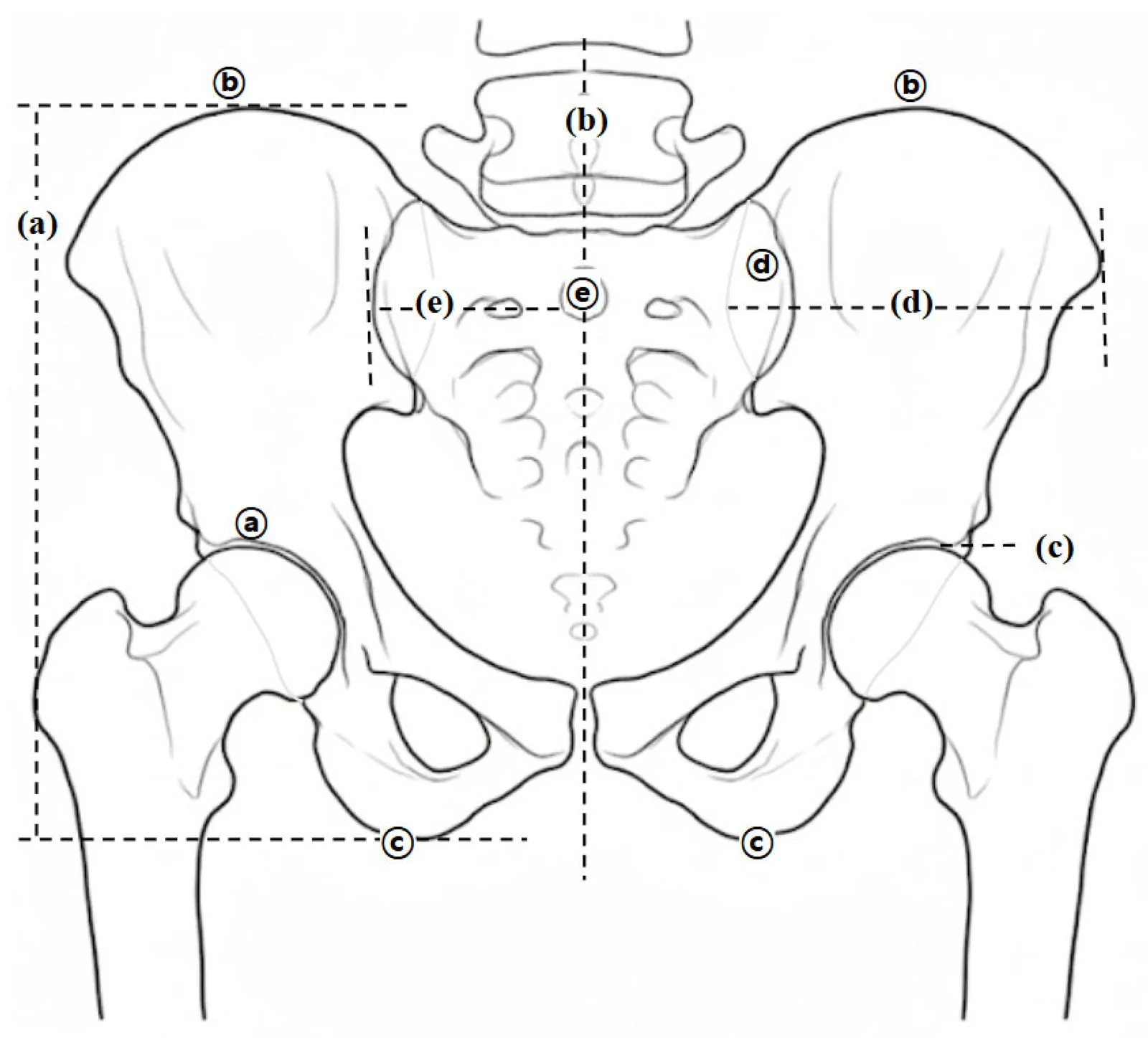
Radiographic indicators for diagnosing pelvic malalignment. (**a**) innominate measurement; (**b**) off-centering measurement; (**c**) femur head line; (**d**) ilium shadow measurement (ISM), the linear distance between the PSIS reference line and the lateral iliac-border reference line, both drawn perpendicular to the femur baseline; (**e**) sacral ala measurement; ⓐ femur base; ⓑ apex of iliac crest; ⓒ ischial tuberosity; ⓓ sacral groove; ⓔ S2 tubercle.

Pelvic alignment was quantitatively assessed by marking anatomical landmarks on standing AP X-ray images using a picture archiving and communication system to assess: (1) innominate rotation and (2) in-flare/out-flare displacement (Figure 2). Given the absence of standardized criteria for non-fracture pelvic malalignment, an expert panel of experienced clinicians established standardized diagnostic thresholds through iterative discussions and calibration. Specifically, these thresholds were defined to reflect clinically meaningful degrees of malalignment that warrant CMT, based on practical clinical experience.

(1) Innominate Rotation: Malalignment was assessed based on the bilateral difference in the innominate measurement (IM), defined as the vertical distance between the horizontal line through the highest point of the iliac crest and the horizontal line through the lowest point of the ischial tuberosity [13,14,22]. Per expert consensus, a difference of ≥3.5 mm was set as the diagnostic threshold. The side with the shorter IM indicated anterior rotation, whereas the longer IM indicated posterior rotation. Diagnostic specificity was further enhanced by referencing the obturator foramen area and femoral head height. In the present study, differences of >85 mm^2^ and >4 mm, respectively, were applied based on expert consensus.
(2) In-flare/Out-flare Displacement: The assessment was based on the bilateral difference in the ilium shadow measurement (ISM), a field-specific Korean Medicine radiographic linear measure defined as the distance between two lines drawn perpendicular to the femur baseline: one passing through the PSIS and the other through the lateral-most radiographic border of the ilium. “Shadow” denotes the radiographic silhouette used to identify the lateral iliac boundary; ISM is the resulting linear distance [2,13,14,22]. A difference of ≥3.0 mm was set as the threshold. A narrower ISM indicated in-flare, whereas a wider ISM indicated out-flare. In ambiguous cases, off-center measurement and PSIS-S2 distance were used to support the diagnosis.

#### 2.4.3. 3D Body Surface Analysis

**Figure 3:**
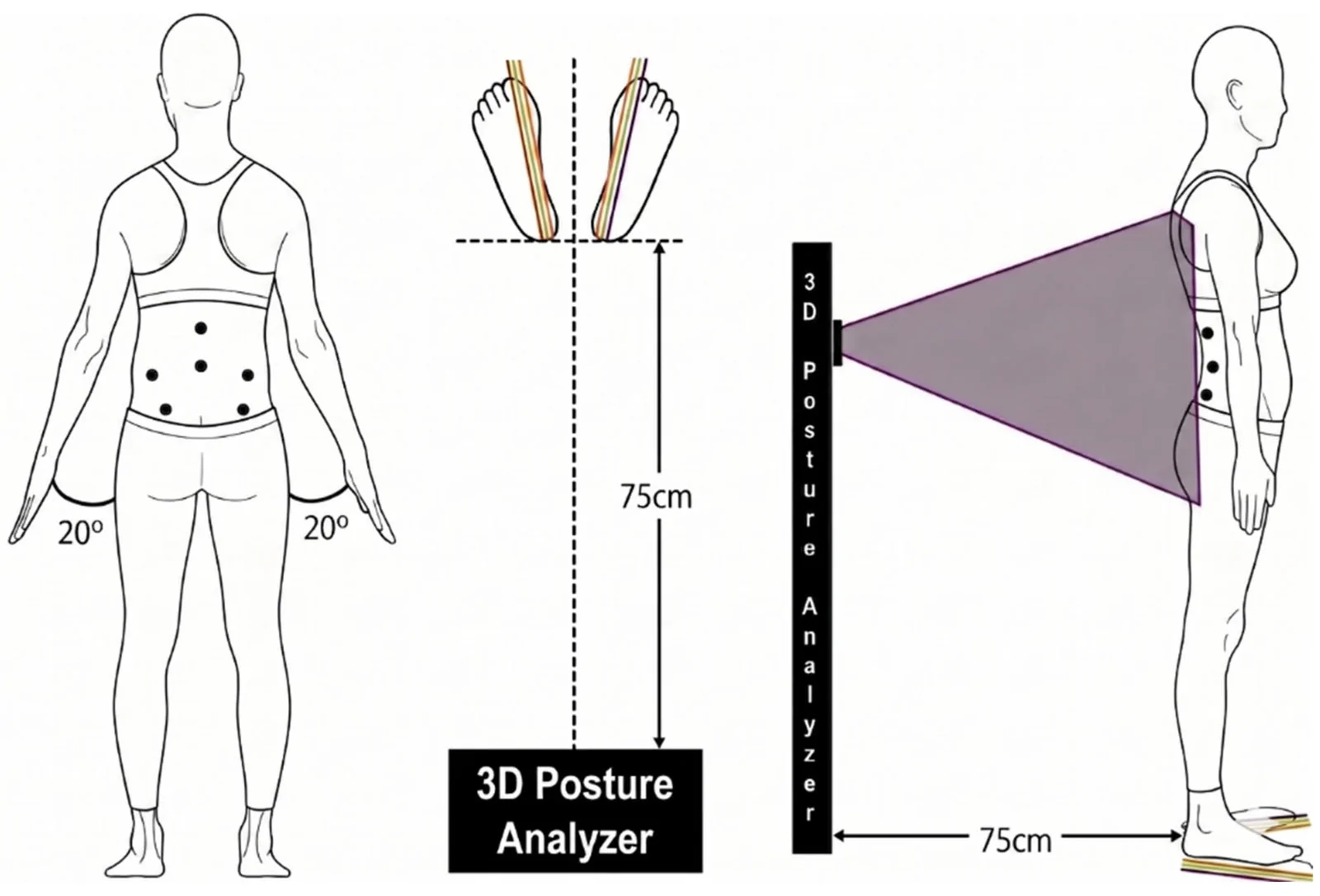
Standardized setup and postural protocol for 3D body surface scanning. Black dots indicate skin markers at L1, L4, the bilateral iliac crests, and the bilateral posterior superior iliac spines. The 20° labels indicate arm abduction, and the 75 cm labels indicate the participant-to-scanner distance. The shaded area schematically illustrates the scanning field.

The 3D analysis used the iBalance system (Team Elysium Inc., Seoul, Republic of Korea) equipped with an Azure Kinect DK (Microsoft Corp., Redmond, WA, USA) (Figure 3). In a controlled experimental evaluation, the Azure Kinect showed systematic spatial errors of less than 2 mm at measurement distances of 1–2 m [23]. To optimize the field of view of the lumbopelvic region and minimize noise, the measurement distance was fixed at 75 cm (Figure 3).

**Figure 4:**
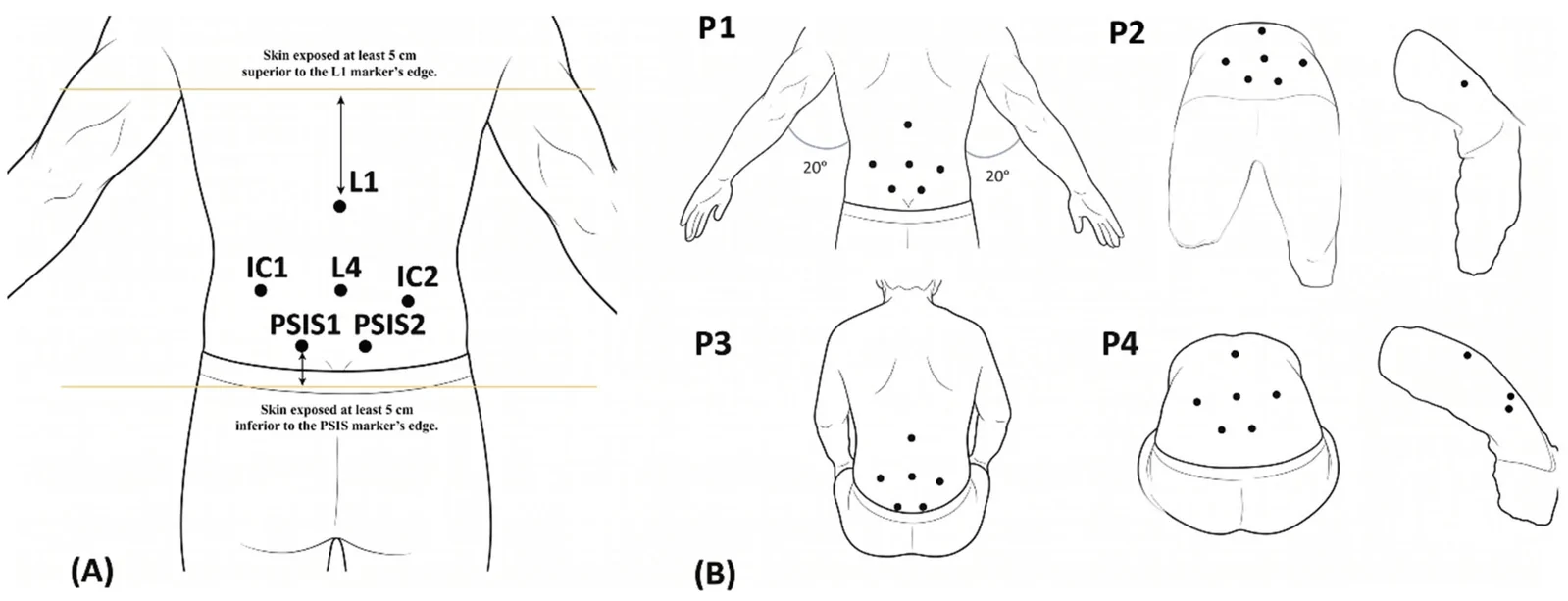
Standardized procedures for 3D body surface analysis. (**A**) Participant-derived schematic illustrating anatomical landmarks and surface marker placement for 3D postural assessment, including L1, L4, bilateral iliac crest (IC), and posterior superior iliac spine (PSIS) markers. The body contour and marker positions reflect the geometry of the original scan and are therefore not intended to represent an idealized symmetric anatomical model. (**B**) Four standardized postures (**P1**–**P4**) used for 3D body surface scanning: standing (**P1**), standing flexion (**P2**), sitting (**P3**), and sitting flexion (**P4**). IC, iliac crest; PSIS, posterior superior iliac spine.

Surface markers were placed on six anatomical landmarks: (1) L1 and L4 spinous processes, (2) bilateral IC, and (3) bilateral PSIS (Figure 4A). An experienced clinician followed the SOP for marker placement described in Section 2.3. For ambiguous landmark placements, a second clinician confirmed the marker location at both institutions. 3D scans were acquired by a clinical research coordinator (CRC) who had no role in the manual examination or X-ray interpretation. Point-cloud data for the PSIS and IC regions were subsequently extracted using an automated algorithm by a researcher at the Korea Institute of Oriental Medicine (KIOM), who was blinded to the manual examination and X-ray findings.

A previous study by members of our research group using the same 3D body surface measurement system evaluated within-session repeatability of selected grid-derived bilateral depth features under three repeated measurements per posture; mean intraclass correlation coefficient (ICC) values ranged from 0.778 to 0.946 across the four postures [24]. This prior evidence does not establish the reliability of the complete protocol used in the present study, including palpation-based landmark identification and marking, the specific PSIS/IC surface-height and marker-angle indicators, single-scan acquisition, and implementation across the two study sites; within-session repeatability, test–retest reliability, inter-rater reliability, and inter-site reproducibility were not directly evaluated in the present study and are identified as priorities for future validation studies. Measurements were obtained in four standardized postures—standing (P1), standing flexion (P2), sitting (P3), and sitting flexion (P4)—to capture functional malalignment (Figure 4B).

### 2.5. Outcome Measures

Outcome measures were evaluated across a prespecified prospective comparison and linked exploratory analyses. The prespecified prospective comparison assessed 3D body surface indicators among participants who completed visit 2 (Section 3.1). The linked exploratory analyses evaluated manual diagnostic reliability and the agreement of X-ray assessment and 3D body surface indicators with final manual diagnosis (Section 3.3). Participant classification criteria differed according to the analytical objective.

#### 2.5.1. Outcomes for the Prespecified Prospective Comparison

This exploratory phase examined whether 3D body surface indicators differed between the protocol-defined malalignment group (*n* = 30) and comparison group (*n* = 30), as described in Section 2.1.

- Primary outcome: Vertical surface-height asymmetry around the bilateral PSIS and IC regions derived from 3D body surface analysis.
- Secondary outcomes: Relative angles between bilateral PSIS and IC markers derived from 3D body surface analysis and bilateral differences in innominate measurement (IM) and ilium shadow measurement (ISM) derived from X-ray assessment.
- Safety evaluation: Vital signs were measured at visits 1 and 2 according to the SOP, and adverse events related to the study procedures were documented.

#### 2.5.2. Outcomes for Linked Exploratory Analyses

The linked exploratory analyses evaluated manual diagnostic reliability and the apparent classification performance of X-ray assessment and 3D body surface analysis relative to final manual diagnosis. Final manual diagnosis was used as an expert-judgment reference comparator rather than as an independently validated diagnostic gold standard. Accordingly, sensitivity, specificity, predictive values, and AUC estimates were interpreted as measures of apparent classification performance relative to expert manual judgment rather than as definitive diagnostic accuracy.

- Manual diagnostic reliability and X-ray agreement: Intra- and inter-rater reliability of manual diagnosis and the apparent classification performance of X-ray assessment relative to final manual diagnosis were calculated among all screened participants (*n* = 88).
- Post-hoc exploratory 3D analysis: Among the same 60 participants who completed 3D measurements, participants were reclassified according to final manual diagnosis alone, yielding manual-positive (*n* = 40) and manual-negative (*n* = 20) groups. Sensitivity, specificity, predictive values, and AUC estimates were calculated for individual 3D indicators and exploratory multivariable models.

### 2.6. Statistical Analysis

Statistical analyses were conducted for three distinct analytical components: (1) the prespecified prospective inter-group comparison among the 60 trial participants; (2) manual diagnostic reliability and X-ray agreement analyses among all 88 screened participants; and (3) post-hoc exploratory ROC analyses of 3D indicators among the same 60 trial participants reclassified according to final manual diagnosis. No missing-data imputation was required. Statistical analyses were performed using SPSS (version 29.0; IBM Corp., Armonk, NY, USA) and MedCalc (version 19.2.6; MedCalc Software Ltd., Ostend, Belgium). A two-sided *p*-value < 0.05 was used as the nominal threshold for statistical significance. Because multiplicity adjustment was not prespecified for the exploratory analyses, *p*-values from these analyses were interpreted as hypothesis-generating rather than confirmatory.

#### 2.6.1. Statistical Methods for the Prespecified Inter-Group Comparison

Participants were analyzed according to the protocol-defined enrollment groups: the malalignment group (*n* = 30) and the comparison group (*n* = 30), as described in Section 2.1. As all trial participants provided complete data, the full analysis set and per-protocol set were identical (full analysis set = per-protocol set = 60).

Continuous variables were compared using independent two-sample *t*-tests or Wilcoxon rank-sum tests after assessment of distributional assumptions. Categorical variables were analyzed using the chi-squared test or Fisher’s exact test, as appropriate. The primary outcome, 3D surface-height asymmetry around the PSIS and IC regions, and the secondary outcomes were evaluated using the same analytical procedures.

#### 2.6.2. Exploratory Agreement Analyses

Final manual diagnosis was used as an expert-judgment reference comparator for the X-ray and 3D exploratory analyses. In the absence of an independently validated diagnostic gold standard for non-fracture pelvic malalignment, the high inter-rater reliability of final manual diagnosis (κ = 0.859) was interpreted as strong agreement among trained examiners applying a standardized SOP, but not as evidence of diagnostic validity or gold-standard status. Accordingly, sensitivity, specificity, predictive values, and AUC estimates were interpreted as measures of apparent classification performance relative to expert manual judgment, rather than as definitive diagnostic accuracy against an independently validated reference standard.

(1) Manual diagnostic reliability and X-ray agreement (*n* = 88): Cohen’s kappa was calculated for intra- and inter-rater reliability of manual diagnosis. Sensitivity, specificity, positive predictive value, negative predictive value, and overall concordance were calculated for X-ray assessment relative to final manual diagnosis.
(2) Post-hoc exploratory analysis of 3D body surface indicators (*n* = 60): ROC curve analyses were conducted among the same 60 participants who completed 3D measurements and were reclassified according to final manual diagnosis alone, yielding manual-positive (*n* = 40) and manual-negative (*n* = 20) groups. This reclassification, ROC analysis, threshold derivation, and multivariable model development were not prespecified. For individual 3D indicators, optimal thresholds were derived post hoc from the study data using the maximum Youden index (sensitivity + specificity − 1). Sensitivity, specificity, positive predictive value, negative predictive value, and AUC were calculated for individual 3D indicators and exploratory multivariable models. The resulting thresholds were data-derived and exploratory and should not be interpreted as validated clinical cut-off values.

As a separate post-hoc sensitivity analysis, an alternative parsimonious multivariable model was explored using the least absolute shrinkage and selection operator (LASSO) to select variables from 16 absolute-value 3D indicators. For this alternative model, the optimal probability threshold was selected post hoc using the maximum Youden index, and bootstrap internal validation with 1000 resamples was used to estimate optimism in apparent model performance. This sensitivity analysis did not validate the original five-variable model. Because participant reclassification, predictor exploration, threshold derivation, and model development were performed post hoc in the same small pilot dataset, all findings should be interpreted as exploratory and hypothesis-generating. Independent external validation is required before any clinical application.

An overview of the analytical datasets, classification frameworks, reference bases, and study objectives is presented in Table 1.

## 3. Results

### 3.1. Prespecified Inter-Group Comparison of Prospective Trial Participants

The prespecified prospective comparison included the protocol-defined malalignment group (*n* = 30) and comparison group (*n* = 30). Baseline demographic and clinical characteristics are presented in Supplementary Table S1.

(1) Primary Outcome: Surface-Height Asymmetry

Comparison of surface-height asymmetry around the PSIS and IC regions, the primary outcome, revealed no statistically significant differences between the malalignment and comparison groups across any posture (P1–P4), including standing (P1) (all *p* > 0.05; Table 2).

(2) Secondary Outcomes: 3D PSIS/IC Marker Angles

Relative angles between bilateral PSIS and IC markers derived from 3D body surface analysis also showed no statistically significant between-group differences across all postures (P1–P4) (*p* > 0.05; Table 3).

(3) Secondary Outcomes: ISM/IM Bilateral Differences (X-ray)

Radiographic structural indicator analysis showed that the bilateral ISM length difference was significantly greater in the malalignment group than that in the comparison group (*p* = 0.028). In contrast, no significant difference was observed in bilateral IM length difference between the two groups (*p* = 0.551; Table 4).

No adverse events related to the study procedures were reported among the 60 trial participants.

### 3.2. Relationship Between Protocol-Defined Enrollment Groups and Post-Hoc Classification

Among the 60 participants who completed 3D measurements, all 30 participants initially assigned to the protocol-defined malalignment group were classified as manual-positive according to final manual diagnosis. Among the 30 participants initially assigned to the comparison group, 10 were classified as manual-positive and 20 as manual-negative. Accordingly, post-hoc reclassification of the same 60 participants according to final manual diagnosis yielded 40 manual-positive and 20 manual-negative participants (Table 5).

### 3.3. Additional Exploratory Agreement Analyses

The following analyses used data from all screened participants (*n* = 88) for manual diagnostic reliability and X-ray agreement, and data from the same 60 participants who completed visit 2 for post-hoc exploratory 3D analyses.

#### 3.3.1. Intra- and Inter-Rater Reliability of Manual Diagnosis

The final pelvic malalignment diagnosis showed almost perfect intra-rater agreement (*κ* = 0.930, 95% CI 0.852–1.000) and inter-rater agreement (*κ* = 0.859, 95% CI 0.750–0.968; *p* < 0.001; Table 6). Reliability for most individual items was high (*κ* ≥ 0.8); however, coefficients for left anterior iliac rotation (LAIR), neutral iliac rotation (NIR), and neutral flare (NF) could not be calculated because of zero variance. This indicates that these specific subtypes were absent in the present sample. Therefore, the overall reliability estimate applies to the malalignment patterns observed in this study and may not generalize to populations with different subtype distributions.

#### 3.3.2. Agreement of X-Ray Assessment with Final Manual Diagnosis

Agreement of X-ray assessment with final manual diagnosis was evaluated using interpretation values obtained during the screening phase, with final manual diagnosis serving as the expert-judgment reference comparator (Table 7). A total of 88 participants who completed both X-ray and manual examinations were included in the analysis. X-ray assessment showed a sensitivity of 88.6%, specificity of 34.1%, positive predictive value (PPV) of 57.4%, negative predictive value (NPV) of 75.0%, and concordance rate of 61.4% relative to final manual diagnosis. In summary, the 2 × 2 cross-tabulation for the overall X-ray final diagnosis versus manual final diagnosis (*n* = 88) was as follows: true positive = 39, false positive = 29, false negative = 5, true negative = 15. Regarding the performance of individual X-ray indicators, RAIR and RPIR showed high specificity (86.4% and 97.7%, respectively) but low sensitivity (18.2% and 15.9%, respectively). NIR exhibited poor overall discriminative ability, with a sensitivity of 40.9% and a specificity of 20.5%. For left pelvic (LP) deviation, no X-ray-positive cases were identified, including among the 44 participants classified as positive by final manual diagnosis; sensitivity was therefore 0% and specificity was 100%.

#### 3.3.3. Exploratory Agreement Analysis of 3D Body Surface Indicators

ROC curve analyses were conducted among the 60 participants who completed 3D measurement and were reclassified according to final manual diagnosis as manual-positive (*n* = 40) or manual-negative (*n* = 20). Final manual diagnosis was used as an expert-judgment reference comparator. Accordingly, the AUC, sensitivity, and specificity estimates represent apparent agreement with expert manual judgment rather than diagnostic accuracy against an independently validated gold standard. The reported operating thresholds were data-derived and should be interpreted as exploratory rather than validated clinical decision thresholds.

In this post-hoc exploratory analysis, several individual 3D indicators showed apparent discrimination. The highest AUC among the individual indicators was observed for the angle between the bilateral IC markers at P2 (AUC = 0.706, *p* = 0.005). IC surface-height difference at P3 also showed apparent discrimination (AUC = 0.684, *p* = 0.015). Because participants were reclassified after enrollment and multiple indicators and postures were explored in the same small dataset, these findings should be interpreted as hypothesis-generating.

**Figure 5:**
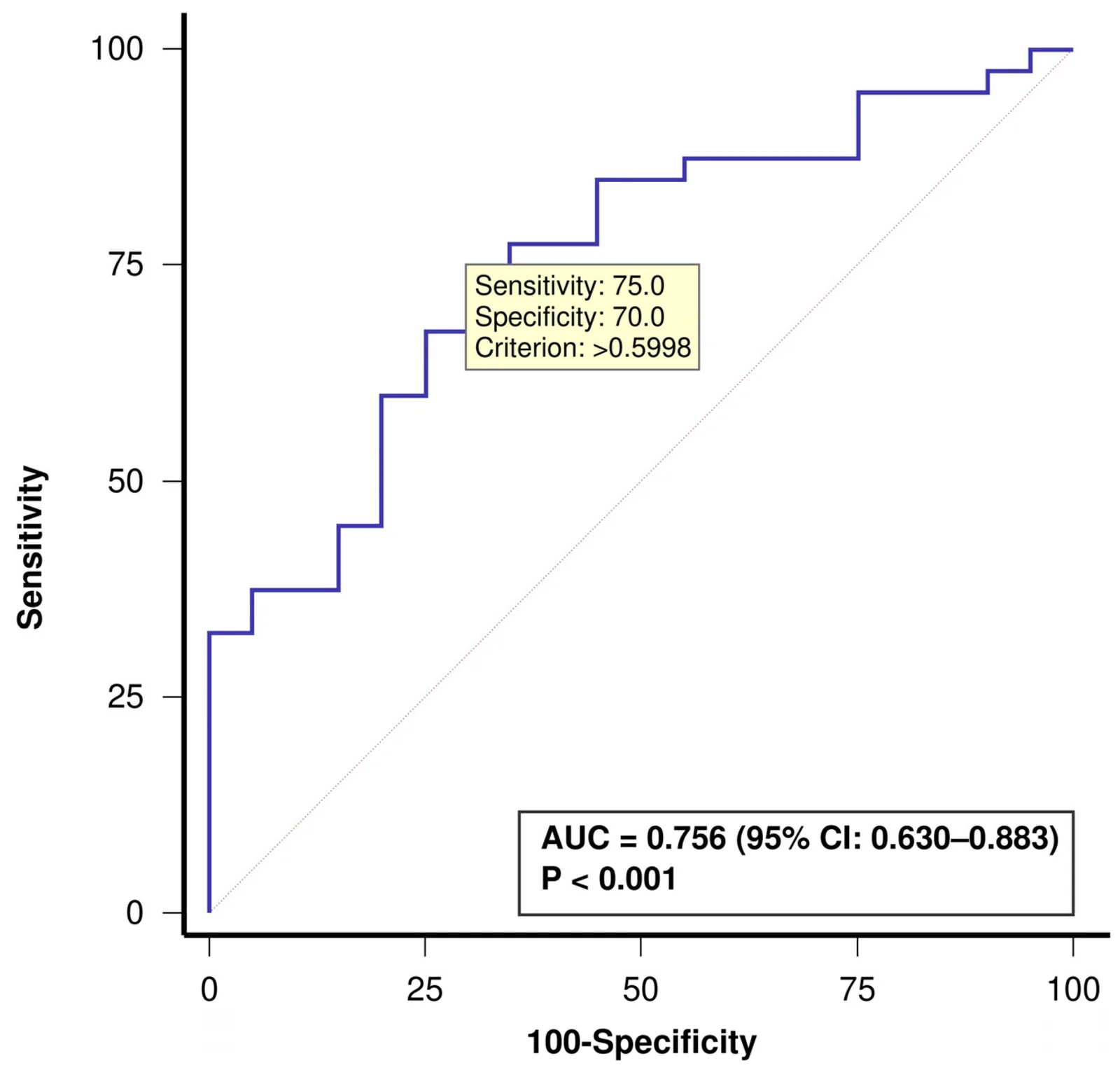
Apparent in-sample ROC curve of the original five-variable multivariable model against final manual diagnosis as an expert-judgment reference comparator among the 60 participants who completed 3D measurement. The operating point shown corresponds to a predicted-probability threshold derived post hoc from the study data. AUC: area under the curve; CI: confidence interval; ROC: receiver operating characteristic.

**Figure 6:**
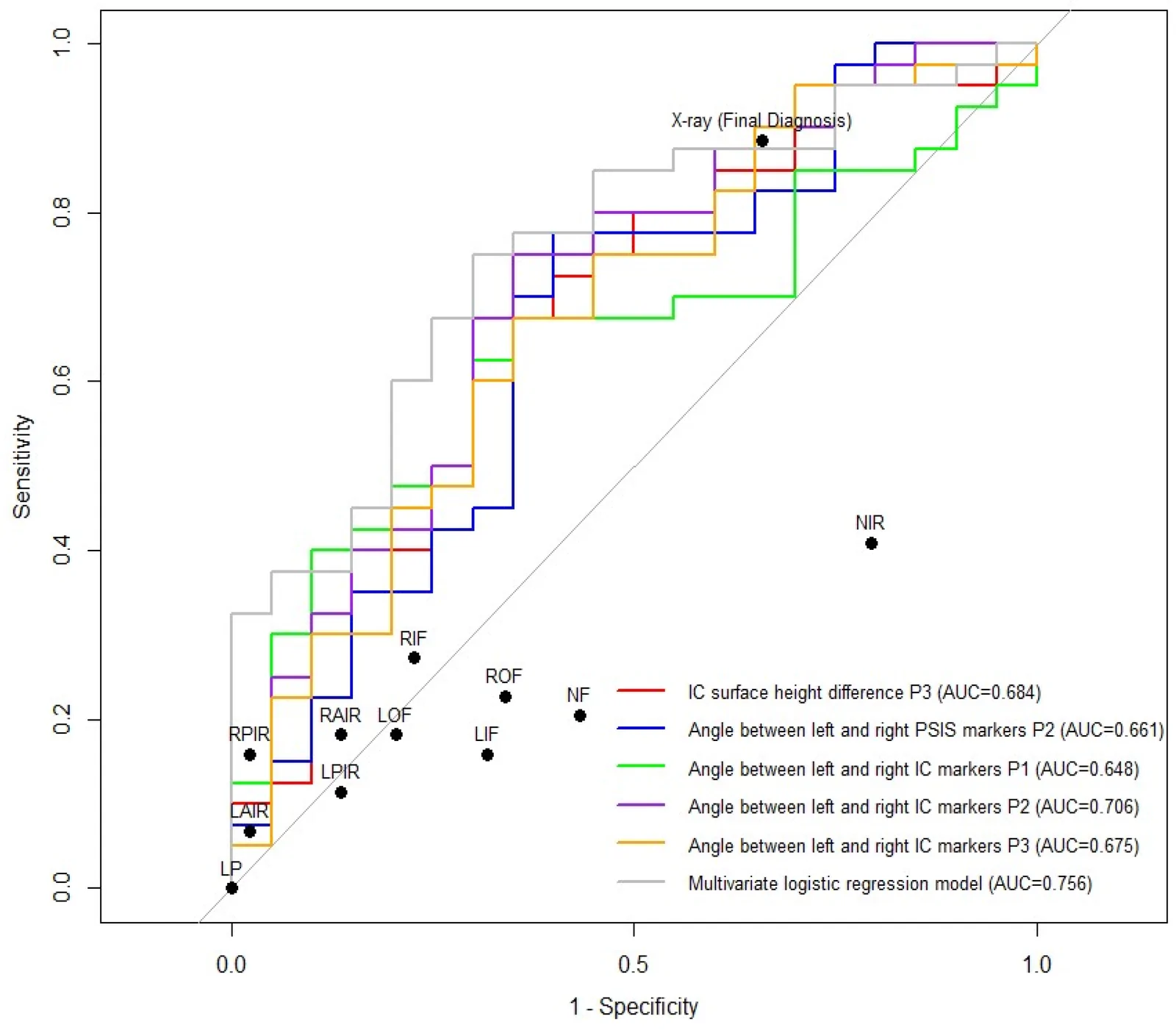
ROC curves of selected 3D body surface indicators and the original five-variable multivariable model against final manual diagnosis as an expert-judgment reference comparator among the 60 participants who completed 3D measurement. Black dots represent the operating points of the X-ray indicators and the final X-ray diagnosis in the screening sample (n = 88), whereas the ROC curves are based on the 60 participants who completed 3D measurements. AUC: area under the curve; IC: iliac crest; LAIR: left anterior iliac rotation; LIF: left in-flare; LOF: left out-flare; LP: left pelvic deviation; LPIR: left posterior iliac rotation; NF: neutral flare; NIR: neutral iliac rotation; PSIS: posterior superior iliac spine; RAIR: right anterior iliac rotation; RIF: right in-flare; ROC: receiver operating characteristic; ROF: right out-flare; RPIR: right posterior iliac rotation. Red: IC surface-height difference at P3; Blue: angle between the bilateral PSIS markers at P2; Green: angle between the bilateral IC markers at P1; Purple: angle between the bilateral IC markers at P2; Orange: angle between the bilateral IC markers at P3; Gray: original five-variable multivariable model.

The original five-variable multivariable model yielded an apparent in-sample AUC of 0.756 (Table 8, Figure 5 and Figure 6). This model was developed and evaluated in the same post-hoc dataset and was not independently validated.

In a separate post-hoc LASSO sensitivity analysis, an alternative parsimonious model yielded an apparent AUC of 0.758. Bootstrap internal validation with 1000 resamples yielded an optimism-corrected AUC of 0.713. This analysis evaluated the alternative model only and did not validate the original five-variable model.

## 4. Discussion

We compared findings from manual examination, X-ray assessment, and 3D body surface analysis in patients with non-acute LBP and explored whether selected 3D indicators showed signals associated with expert manual judgment that might warrant further investigation.

### 4.1. Interpretation of the Prespecified 3D Comparisons

In the prespecified prospective comparison of the initial enrollment groups, neither the primary outcome—surface-height difference around the PSIS and IC regions—nor the secondary marker-angle outcome differed significantly between groups across postures P1–P4. Several explanations are possible, none of which can be distinguished with the present data. Vertical (z-axis) surface information may have been insufficiently sensitive to the differences between the initial enrollment groups; the distance- and position-dependent measurement error of the Azure Kinect DK sensor [23], soft-tissue characteristics over palpated bony landmarks [25,26], and other unmeasured sources of protocol-level variability may have reduced the detectability of small surface differences; and the protocol-defined composition of the comparison group may have reduced the contrast between the two enrollment groups. Because measurement error was not quantified in this study, these remain hypotheses rather than demonstrated causes.

Both the surface-height and marker-angle outcomes were non-significant in the prespecified comparison of the initial enrollment groups. In a separate post-hoc ROC analysis, in which the 60 participants were reclassified according to final manual diagnosis, the highest AUC among the individual 3D indicators was observed for the angle between the bilateral IC markers at P2. Because the two analyses used different classification frameworks and the latter involved post-hoc exploration of multiple indicators and thresholds, this finding does not establish that angle measures outperform surface-height measures.

A rationale that could be tested in future work is that angle-based measures encode the relative orientation between two landmarks rather than absolute depth at a single point, and may therefore behave differently under local soft-tissue variation. This rationale was not tested in the present study, and the data reported here do not evaluate it. Prospective designation of angle-based indicators, with prespecified thresholds and formal reliability assessment, would be required before any such property could be claimed.

### 4.2. Discordance Between Modalities

The X-ray and 3D analyses examined in this study were evaluated against an expert-judgment manual comparator and should not be interpreted as direct substitutes for that comparator. In the screening sample, X-ray showed high sensitivity but low specificity relative to final manual diagnosis, and this asymmetry warrants interpretation. Manual examination is applied clinically to identify functional features such as segmental hypomobility [27] and asymmetric excursion during motion, rather than static anatomical geometry alone; it is widely used in this clinical setting and has been evaluated alongside radiographic and AI-assisted diagnostic methods [4], although its criterion validity against independent reference standards remains limited [10]. High inter-rater agreement (Table 6) indicates that trained examiners applied the standardized protocol consistently; it does not establish that manual diagnosis captures the underlying alignment state. Manual diagnosis is therefore treated throughout as an expert-judgment comparator, and the agreement statistics reported here should be read accordingly.

Low specificity relative to the manual comparator may reflect discordance between the structural measures obtained radiographically and the functional features targeted by manual assessment. It may also reflect imperfection of the manual comparator itself. The present design cannot distinguish between these explanations, and the reported values should not be read as evidence that either modality is in error.

ISM was the only radiographic indicator that differed between the initial enrollment groups. This isolated finding, obtained without prespecified multiplicity adjustment, requires replication and does not establish ISM as a preferred or bridging measure; the clinical relevance of the small between-group difference was not evaluated in this study. The original five-variable multivariable model was developed and evaluated in the same post-hoc dataset. A separate post-hoc LASSO-based sensitivity analysis yielded a similar exploratory level of discrimination but did not validate the original multivariable model or establish incremental clinical value. Any clinical role for 3D body surface analysis therefore remains undetermined. On present evidence, it can be positioned only as a candidate non-radiographic adjunct to expert manual assessment requiring prospective validation; longitudinal monitoring of alignment change during treatment was not evaluated in this study and would require dedicated repeatability and responsiveness studies.

### 4.3. Limitations and Future Directions

This exploratory pilot study has several methodological limitations that should be considered when interpreting the findings. They fall into three distinct categories: the status of the reference comparator and participant selection, the reuse of a single sample for model development and evaluation, and the absence of formal reliability assessment.

(1) Reference comparator and participant selection

Manual diagnosis is not an independently validated gold standard for non-fracture pelvic malalignment. High inter-rater agreement under a standardized SOP indicates consistent application of shared clinical criteria, but does not guarantee that those criteria capture the underlying alignment state. Manual diagnosis was used both to help define the prospective enrollment groups and as the comparator for the X-ray and 3D exploratory analyses; this overlapping use may have introduced dependence between the enrollment classification and the manual comparator, thereby complicating interpretation of the subsequent X-ray and 3D analyses. In addition, the protocol-defined comparison group comprised participants in the definite-negative category and participants allocated to the protocol-defined subgroup-analysis category. This heterogeneous composition may have reduced contrast between the enrollment groups and limited the ability of the prespecified 30-versus-30 comparison to detect between-group differences. Because 3D scanning was confined to participants selected through the study-specific screening and enrollment algorithm, the enrolled sample may not represent the full spectrum of patients with non-acute LBP; the direction and magnitude of any selection-related bias cannot be estimated from these data.

In addition, no formal statistical adjustment for potential confounding variables, such as age, sex, and body mass index, was prespecified or performed in this exploratory pilot analysis. Participant characteristics were restricted where specified by the eligibility criteria and reported descriptively in Supplementary Table S1; therefore, the reported exploratory associations should not be interpreted as adjusted or confounder-controlled estimates.

(2) Model development and sample

Distinct from the circularity described above, a separate source of bias arises from the reuse of the same *n* = 60 sample. Post-hoc reclassification, candidate-indicator exploration across four postures, threshold derivation, and model fitting and evaluation were all conducted within this single dataset, without prespecified correction for multiplicity and without an independent validation sample [28]. The reported operating characteristics are therefore apparent in-sample estimates and are expected to be optimistic relative to an independent sample. The five-variable model had an events-per-variable ratio of 8 (40 manual-positive participants and five model predictors), which is below ratios commonly cited as desirable in prediction-model development and may have contributed to unstable estimates. The small number of manual-negative participants further limits the precision of model-performance estimates. The overall fit of the original five-variable model was not statistically significant, and its coefficient estimates were unstable, further limiting interpretation of this model as a predictive model. A separate post-hoc analysis of an alternative parsimonious model derived by LASSO evaluated that alternative model only; it did not validate the five-variable model and does not substitute for validation in an independent sample. Finally, no formal a priori power calculation was performed; the target of 30 participants per group was chosen on feasibility grounds, and the study was not powered for confirmatory hypothesis testing.

(3) Measurement and inter-site reliability

Pelvic X-rays were interpreted by one designated reader at each institution, that is, by different readers across the two sites, using a shared SOP. During the study period, one reader was replaced following completion of residency training. Inter-site reader variability was not formally quantified through a cross-site reliability check, and may have contributed to the observed radiographic performance. For the 3D protocol, landmark determination depends on manual palpation; despite the standardized placement guideline applied identically at both sites, residual variability related to palpation-based marker placement, skin-surface characteristics, and posture acquisition cannot be excluded [26]. Direct quantification of these sources of variability was not undertaken in the present study; additional radiographic imaging for this purpose was not ethically justifiable, and non-radiographic reliability substudies were not part of the trial protocol. Landmark placement followed the standardized SOP, and ambiguous locations were confirmed by a second clinician; however, these procedures do not replace formal reliability assessment. Each participant underwent a single scan per posture, so within-session repeatability, test–retest reliability, inter-rater reliability, and inter-site reproducibility of the complete acquisition protocol were not assessed in the present study.

A previous study by members of our research group using the same measurement system reported within-session repeatability of selected grid-derived bilateral depth features under repeated acquisition [24]; however, these results cannot be extrapolated directly to the single-scan PSIS/IC surface-height and marker-angle indicators used here. In that previous grid-based analysis, standing flexion showed the lowest repeatability among the four postures. This posture-specific finding cannot be extrapolated to the P2 IC marker-angle measure identified in the present post-hoc analysis.

A separate automated landmark-localization study using pelvic surface scans evaluated within-visit repeatability of paired landmark distances; in that separate dataset, automated localization showed higher repeatability than manual landmark marking [29]. That work did not validate the landmark-identification or measurement protocol used in the present study.

Future studies should use prospectively defined recruitment criteria that are independent of the index-test evaluation, prespecify candidate indicators and thresholds before analysis, adopt an independent or composite reference standard where feasible, and validate performance in independent samples.

## 5. Conclusions

In this exploratory pilot study, the prespecified prospective comparison did not identify significant differences in either the primary 3D surface-height outcome or the secondary marker-angle outcome between the protocol-defined enrollment groups. In separate post-hoc analyses of the same 60 participants reclassified according to final manual diagnosis, several 3D indicators showed apparent associations with expert manual judgment. Because these findings arose from post-hoc reclassification, exploration of multiple indicators and thresholds, and evaluation within the same small dataset, they should be regarded as hypothesis-generating rather than as evidence of diagnostic accuracy or clinical utility. Accordingly, 3D body surface analysis should not currently be regarded as a stand-alone diagnostic or screening tool. Prospective studies with prespecified indicators and thresholds, formal reliability assessment, an appropriate independent or composite reference standard, and independent validation are required before any clinical role for 3D body surface analysis can be established.

## Figures and Tables

**Table 1:** Overview of analytical datasets, classification frameworks, reference bases, and study objectives.

Analysis	Participants	Classification Framework/Reference Basis	Objective	Analytical Status
Prespecified prospective inter-group comparison	60 (30 vs. 30)	Protocol-defined enrollment algorithm based on screening manual examination and X-ray findings	Comparison of prespecified 3D surface-height and marker-angle outcomes	Prespecified
Manual diagnostic reliability	88	Independent manual assessments by two examiners using the standardized SOP	Assessment of intra- and inter-rater reliability of manual diagnosis	Exploratory secondary analysis
X-ray agreement with final manual diagnosis	88	Final manual diagnosis as an expert-judgment reference comparator	Assessment of apparent classification performance of X-ray findings relative to expert manual judgment	Exploratory secondary analysis
Post-hoc 3D ROC analysis	Same 60 participants (40 manual-positive vs. 20 manual-negative)	Reclassification according to final manual diagnosis alone	Exploration of apparent discrimination of candidate 3D indicators relative to expert manual judgment	Post-hoc exploratory

**Table 2:** Differences in the primary outcome: surface height asymmetry around PSIS and IC.

Variable (*n* = 60)	Posture	Group	Mean ± SD (mm)	*p*-Value
Surface height difference around PSIS	P1	Malalignment	−3.2 ± 6.3	0.427
		Comparison group	−1.9 ± 5.9	
	P2	Malalignment	−1.8 ± 5.2	0.371
		Comparison group	−0.7 ± 3.9	
	P3	Malalignment	−1.4 ± 5.9	0.955
		Comparison group	−1.3 ± 5.8	
	P4	Malalignment	−1.2 ± 4.1	0.523
		Comparison group	−0.4 ± 5.7	
Surface height difference around IC	P1	Malalignment	−4.1 ± 7.2	0.356
		Comparison group	−2.4 ± 6.8	
	P2	Malalignment	−2.1 ± 6.1	0.365
		Comparison group	−0.8 ± 4.8	
	P3	Malalignment	−2.2 ± 7.3	0.846
		Comparison group	−1.8 ± 6.7	
	P4	Malalignment	−1.7 ± 5.0	0.425
		Comparison group	−0.5 ± 6.8	

IC: iliac crest; P: posture; PSIS: posterior superior iliac spine; SD: standard deviation.

**Table 3:** Differences in the secondary outcome: angle between left and right PSIS and IC marker positions.

Variable (*n* = 60)	Posture	Group	Mean ± SD (°)	*p*-Value
Angle between left and right PSIS markers	P1	Malalignment	1.4708 ± 2.8921	0.604
		Comparison group	1.0773 ± 2.9553	
	P2	Malalignment	0.8298 ± 2.6336	0.543
		Comparison group	0.4530 ± 2.1092	
	P3	Malalignment	0.6027 ± 2.6613	0.783
		Comparison group	0.8107 ± 3.1385	
	P4	Malalignment	0.4471 ± 1.8936	0.911
		Comparison group	0.3753 ± 2.9454	
Angle between left and right IC markers	P1	Malalignment	1.8954 ± 3.4312	0.602
		Comparison group	1.4393 ± 3.3081	
	P2	Malalignment	1.1652 ± 3.2285	0.487
		Comparison group	0.6320 ± 2.6515	
	P3	Malalignment	1.0361 ± 3.2733	0.938
		Comparison group	1.1046 ± 3.5103	
	P4	Malalignment	0.7407 ± 2.3201	0.883
		Comparison group	0.6273 ± 3.4860	

IC: iliac crest; P: posture; PSIS: posterior superior iliac spine; SD: standard deviation.

**Table 4:** Differences in secondary outcome: left–right difference in IM and ISM length.

Variable (*n* = 60)	Group	Mean ± SD (mm)	*p*-Value
Left–right difference in IM length	Malalignment	3.01 ± 2.66	0.551
	Comparison group	2.65 ± 1.98	
Left–right difference in ISM length	Malalignment	7.23 ± 4.39	0.028
	Comparison group	4.68 ± 4.38	

IM: innominate measurement; ISM: ilium shadow measurement; SD: standard deviation.

**Table 5:** Relationship between protocol-defined enrollment groups and post-hoc classification according to final manual diagnosis.

Initial Protocol-Defined Enrollment Group	Manual-Positive	Manual-Negative	Total
Malalignment group	30	0	30
Comparison group	10	20	30
Total	40	20	60

**Table 6:** Intra- and inter-rater reliability of manual diagnosis (Cohen’s kappa, 95% CI, *p*).

Variable	Intra-Rater Reliability			Inter-Rater Reliability		
	Kappa	95% CI	*p*-Value	Kappa	95% CI	*p*-Value
RAIR	0.917	0.756–1.000	<0.001	0.821	0.579–1.000	<0.001
RPIR	0.941	0.826–1.000	<0.001	0.823	0.628–1.000	<0.001
LAIR	-*	-	-	-*	-	-
LPIR	1.000	1.000–1.000	<0.001	0.794	0.402–1.000	<0.001
NIR	-*	-	-	-*	-	-
RIF	0.924	0.819–1.000	<0.001	0.883	0.752–1.000	<0.001
ROF	1.000	1.000–1.000	<0.001	0.935	0.808–1.000	<0.001
LIF	1.000	1.000–1.000	<0.001	1.000	1.000–1.000	<0.001
LOF	0.917	0.756–1.000	<0.001	0.845	0.634–1.000	<0.001
NF	-*	-	-	-*	-	-
LP	1.000	1.000–1.000	<0.001	0.662	0.041–1.000	<0.001
Manual diagnosis (Final diagnosis)	0.930	0.852–1.000	<0.001	0.859	0.750–0.968	<0.001

* The kappa statistic could not be calculated because both raters assigned the same value (0). CI: confidence interval; LAIR: left anterior iliac rotation; LIF: left in-flare; LOF: left out-flare; LP: left pelvic deviation; LPIR: left posterior iliac rotation; Manual diagnosis: final diagnosis; NF: neutral flare; NIR: neutral iliac rotation; RAIR: right anterior iliac rotation; RIF: right in-flare; ROF: right out-flare; RPIR: right posterior iliac rotation.

**Table 7:** Agreement of X-ray assessment with final manual diagnosis (*n* = 88): sensitivity, specificity, PPV, NPV, and concordance rate.

Evaluation		Manual Final Diagnosis		Sensitivity, % (95% CI)	Specificity, % (95% CI)	PPV, % (95% CI)	NPV, % (95% CI)	Concordance Rate, %
		Positive (*n* = 44)	Negative (*n* = 44)					
RAIR	1	8	6	18.2 (8.2–32.7)	86.4 (72.6–94.8)	57.1 (28.9–82.3)	51.4 (39.4–63.1)	52.3%
	0	36	38					
RPIR	1	7	1	15.9 (6.6–30.1)	97.7 (88.0–99.9)	87.5 (47.3–99.7)	53.8 (42.2–65.0)	56.8%
	0	37	43					
LAIR	1	3	1	6.8 (1.4–18.7)	97.7 (88.0–99.9)	75.0 (19.4–99.4)	51.2 (40.0–62.3)	52.3%
	0	41	43					
LPIR	1	5	6	11.4 (3.8–24.6)	86.4 (72.6–94.8)	45.5 (16.7–76.6)	49.4 (37.8–61.0)	48.9%
	0	39	38					
NIR	1	18	35	40.9 (26.3–56.8)	20.5 (9.8–35.3)	34.0 (21.5–48.3)	25.7 (12.5–43.3)	30.7%
	0	26	9					
RIF	1	12	10	27.3 (15.0–42.8)	77.3 (62.2–88.5)	54.5 (32.2–75.6)	51.5 (38.9–64.0)	52.3%
	0	32	34					
ROF	1	10	15	22.7 (11.5–37.8)	65.9 (50.1–79.5)	40.0 (21.1–61.3)	46.0 (33.4–59.1)	44.3%
	0	34	29					
LIF	1	7	14	15.9 (6.6–30.1)	68.2 (52.4–81.4)	33.3 (14.6–57.0)	44.8 (32.6–57.4)	42.0%
	0	37	30					
LOF	1	8	9	18.2 (8.2–32.7)	79.5 (64.7–90.2)	47.1 (23.0–72.2)	49.3 (37.2–61.4)	48.9%
	0	36	35					
NF	1	9	19	20.5 (9.8–35.3)	56.8 (41.0–71.7)	32.1 (15.9–52.4)	41.7 (29.1–55.1)	38.6%
	0	35	25					
LP	1	0	0	0.0 (0.0–8.0)	100.0 (92.0–100.0)	-	50.0 (39.1–60.9)	50.0%
	0	44	44					
X-ray (Final diagnosis)	1	39	29	88.6 (75.4–96.2)	34.1 (20.5–49.9)	57.4 (44.8–69.3)	75.0 (50.9–91.3)	61.4%
	0	5	15					

CI: confidence interval; LAIR: left anterior iliac rotation; LIF: left in-flare; LOF: left out-flare; LP: left pelvic deviation; LPIR: left posterior iliac rotation; Manual diagnosis: final diagnosis; NF: neutral flare; NIR: neutral iliac rotation; NPV: negative predictive value; PPV: positive predictive value; RAIR: right anterior iliac rotation; RIF: right in-flare; ROF: right out-flare; RPIR: right posterior iliac rotation; 1: X-ray positive; 0: X-ray negative. Data are presented as percentages with exact binomial (Clopper–Pearson) 95% confidence intervals. Sensitivity, specificity, PPV, and NPV were calculated from the corresponding 2 × 2 contingency tables. Final manual diagnosis served as an expert-judgment reference comparator rather than an independently validated gold standard.

**Table 8:** Post-hoc exploratory agreement of 3D body surface indicators with final manual diagnosis among participants who completed 3D measurement (*n* = 60): AUC, sensitivity, specificity, PPV, and NPV.

3D Examination (*n* = 60)		Cut-Off Value	Manual Final Diagnosis		Cut-off Value	AUC (*p*)	Sensitivity, % (95% CI)	Specificity, % (95% CI)	PPV, % (95% CI)	NPV, % (95% CI)
			1 (*n* = 40)	0 (*n* = 20)						
Surface height difference around PSIS region	P1	>4.7	24 (60.0%)	8 (40.0%)	>4.7	0.565 (0.411)	60.0 (43.3–75.1)	60.0 (36.1–80.9)	75.0 (56.6–88.5)	42.9 (24.5–62.8)
		≤ 4.7	16 (40.0%)	12 (60.0%)						
	P2	>1.6	30 (75.0%)	9 (45.0%)	>1.6	0.612 (0.154)	75.0 (58.8–87.3)	55.0 (31.5–76.9)	76.9 (60.7–88.9)	52.4 (29.8–74.3)
		≤1.6	10 (25.0%)	11 (55.0%)						
	P3	>4.1	20 (50.0%)	6 (30.0%)	>4.1	0.595 (0.216)	50.0 (33.8–66.2)	70.0 (45.7–88.1)	76.9 (56.4–91.0)	41.2 (24.6–59.3)
		≤4.1	20 (50.0%)	14 (70.0%)						
	P4	>1.7	29 (72.5%)	19 (95.0%)	>1.7	0.516 (0.834)	72.5 (56.1–85.4)	5.0 (0.1–24.9)	60.4 (45.3–74.2)	8.3 (0.2–38.5)
		≤1.7	11 (27.5%)	1 (5.0%)						
	Δ P2-1	≤4.7	25 (62.5%)	7 (35.0%)	≤ 4.7	0.604 (0.181)	62.5 (45.8–77.3)	65.0 (40.8–84.6)	78.1 (60.0–90.7)	46.4 (27.5–66.1)
		>4.7	15 (37.5%)	13 (65.0%)						
	Δ P4-3	≤3.6	25 (62.5%)	15 (75.0%)	≤3.6	0.511 (0.890)	62.5 (45.8–77.3)	25.0 (8.7–49.1)	62.5 (45.8–77.3)	25.0 (8.7–49.1)
		>3.6	15 (37.5%)	5 (25.0%)						
Surface height difference around IC region	P1	>5.6	21 (52.5%)	5 (25.0%)	>5.6	0.626 (0.087)	52.5 (36.1–68.5)	75.0 (50.9–91.3)	80.8 (60.6–93.4)	44.1 (27.2–62.1)
		≤5.6	19 (47.5%)	15 (75.0%)						
	P2	>1.3	35 (87.5%)	11 (55.0%)	>1.3	0.644 (0.071)	87.5 (73.2–95.8)	45.0 (23.1–68.5)	76.1 (61.2–87.4)	64.3 (35.1–87.2)
		≤1.3	5 (12.5%)	9 (45.0%)						
	P3	>4.3	27 (67.5%)	6 (30.0%)	>4.3	0.684 (0.015)	67.5 (50.9–81.4)	70.0 (45.7–88.1)	81.8 (64.5–93.0)	51.9 (31.9–71.3)
		≤4.3	13 (32.5%)	14 (70.0%)						
	P4	>3.1	29 (72.5%)	11 (55.0%)	>3.1	0.561 (0.438)	72.5 (56.1–85.4)	45.0 (23.1–68.5)	72.5 (56.1–85.4)	45.0 (23.1–68.5)
		≤3.1	11 (27.5%)	9 (45.0%)						
	Δ P2-1	≤3.1	15 (37.5%)	3 (15.0%)	≤ 3.1	0.591 (0.247)	37.5 (22.7–54.2)	85.0 (62.1–96.8)	83.3 (58.6–96.4)	40.5 (25.6–56.7)
		>3.1	25 (62.5%)	17 (85.0%)						
	Δ P4-3	≤3.3	25 (62.5%)	10 (50.0%)	≤3.3	0.517 (0.834)	62.5 (45.8–77.3)	50.0 (27.2–72.8)	71.4 (53.7–85.4)	40.0 (21.1–61.3)
		>3.3	15 (37.5%)	10 (50.0%)						
Angle between left and right PSIS markers	P1	>2.9546	18 (45.0%)	3 (15.0%)	>2.9546	0.585 (0.260)	45.0 (29.3–61.5)	85.0 (62.1–96.8)	85.7 (63.7–97.0)	43.6 (27.8–60.4)
		≤2.9546	22 (55.0%)	17 (85.0%)						
	P2	>0.6985	31 (77.5%)	8 (40.0%)	>0.6985	0.661 (0.040)	77.5 (61.5–89.2)	60.0 (36.1–80.9)	79.5 (63.5–90.7)	57.1 (34.0–78.2)
		≤0.6985	9 (22.5%)	12 (60.0%)						
	P3	>2.4766	14 (35.0%)	2 (10.0%)	>2.4766	0.597 (0.202)	35.0 (20.6–51.7)	90.0 (68.3–98.8)	87.5 (61.7–98.4)	40.9 (26.3–56.8)
		≤2.4766	26 (65.0%)	18 (90.0%)						
	P4	≤0.2851	8 (20.0%)	0 (0.0%)	≤0.2851	0.511 (0.882)	20.0 (9.1–35.6)	100.0 (83.2–100.0)	100.0 (63.1–100.0)	38.5 (25.3–53.0)
		>0.2851	32 (80.0%)	20 (100.0%)						
	Δ P2-1	≤1.3595	17 (42.5%)	4 (20.0%)	≤1.3595	0.550 (0.520)	42.5 (27.0–59.1)	80.0 (56.3–94.3)	81.0 (58.1–94.6)	41.0 (25.6–57.9)
		>1.3595	23 (57.5%)	16 (80.0%)						
	Δ P4-3	>0.4771	32 (80.0%)	12 (60.0%)	>0.4771	0.529 (0.732)	80.0 (64.4–90.9)	40.0 (19.1–63.9)	72.7 (57.2–85.0)	50.0 (24.7–75.3)
		≤0.4771	8 (20.0%)	8 (40.0%)						
Angle between left and right IC markers	P1	>2.0965	27 (67.5%)	7 (35.0%)	>2.0965	0.648 (0.042)	67.5 (50.9–81.4)	65.0 (40.8–84.6)	79.4 (62.1–91.3)	50.0 (29.9–70.1)
		≤2.0965	13 (32.5%)	13 (65.0%)						
	P2	>1.3424	30 (75.0%)	7 (35.0%)	>1.3424	0.706 (0.005)	75.0 (58.8–87.3)	65.0 (40.8–84.6)	81.1 (64.8–92.0)	56.5 (34.5–76.8)
		≤1.3424	10 (25.0%)	13 (65.0%)						
	P3	>1.882	27 (67.5%)	7 (35.0%)	>1.882	0.675 (0.021)	67.5 (50.9–81.4)	65.0 (40.8–84.6)	79.4 (62.1–91.3)	50.0 (29.9–70.1)
		≤ 1.882	13 (32.5%)	13 (65.0%)						
	P4	>3.2971	13 (32.5%)	3 (15.0%)	>3.2971	0.545 (0.578)	32.5 (18.6–49.1)	85.0 (62.1–96.8)	81.2 (54.4–96.0)	38.6 (24.4–54.5)
		≤3.2971	27 (67.5%)	17 (85.0%)						
	Δ P2-1	≤3.581	31 (77.5%)	12 (60.0%)	≤3.581	0.524 (0.774)	77.5 (61.5–89.2)	40.0 (19.1–63.9)	72.1 (56.3–84.7)	47.1 (23.0–72.2)
		>3.581	9 (22.5%)	8 (40.0%)						
	Δ P4-3	>0.466	34 (85.0%)	13 (65.0%)	>0.466	0.518 (0.840)	85.0 (70.2–94.3)	35.0 (15.4–59.2)	72.3 (57.4–84.4)	53.8 (25.1–80.8)
		≤0.466	6 (15.0%)	7 (35.0%)						
Multivariable logistic regression model		>0.5998	30 (75.0%)	6 (30.0%)	>0.5998	0.756 (<0.001)	75.0 (58.8–87.3)	70.0 (45.7–88.1)	83.3 (67.2–93.6)	58.3 (36.6–77.9)
		≤0.5998	10 (25.0%)	14 (70.0%)						

AUC: area under the curve; CI: confidence interval; IC: iliac crest; NPV: negative predictive value; P: posture; PPV: positive predictive value; PSIS: posterior superior iliac spine; ΔP2–1 or 4–3: postural change; 1: manual-positive; 0: manual-negative. Surface-height differences are expressed in millimeters, marker angles in degrees, and the multivariable model cut-off as a predicted probability. Data are presented as percentages with exact binomial (Clopper–Pearson) 95% confidence intervals. Final manual diagnosis served as an expert-judgment reference comparator rather than an independently validated gold standard. Estimates represent apparent in-sample agreement with expert manual judgment and should not be interpreted as validated clinical decision thresholds.

## Data Availability

The data presented in this study are available on request from the corresponding author due to ethical and privacy restrictions, as the dataset contains identifiable clinical information from human participants collected under institutional ethics approval (IRB No. PNUKHIRB 2022-04-001, JASENG 2022-04-011, and PNUKHIRB 2025-12-005).

## Supplementary Materials

The following supporting information can be downloaded at https://www.mdpi.com/article/10.3390/diagnostics16203293/s1. Table S1: Baseline demographic and clinical characteristics of participants (N = 60). Table S2: STARD 2015 checklist.

## Abbreviations

The following abbreviations are used in this manuscript:

3D	Three-dimensional
AP	Anteroposterior
ASIS	Anterior superior iliac spine
AUC	Area under the curve
BMI	Body mass index
CI	Confidence interval
CMT	Chuna manual therapy
IC	Iliac crest
ICC	Intraclass correlation coefficient
IM	Innominate measurement
IRB	Institutional Review Board
ISM	Ilium shadow measurement
LAIR	Left anterior iliac rotation
LASSO	Least absolute shrinkage and selection operator
LBP	Low back pain
LP	Left pelvic deviation
NF	Neutral flare
NIR	Neutral iliac rotation
NPV	Negative predictive value
NRS	Numeric rating scale
PPV	Positive predictive value
PSIS	Posterior superior iliac spine
ROC	Receiver operating characteristic
SOP	Standard operating procedure

## References

[1] Park T.Y., Moon T.W., Cho D.C., Lee J.H., Ko Y.S., Hwang E.H., Heo K.H., Choi T.Y., Shin B.C. (2014). An introduction to Chuna manual medicine in Korea: History, insurance coverage, education, and clinical research in Korean literature. Integr. Med. Res..

[2] Korean Society of Chuna Manual Medicine for Spine and Nerves (2017). Chuna Manual Medicine.

[3] Kim M.Y., Ha I.H., Lee J.H., Kim J.H., Jung B. (2019). Usage report of Chuna manual therapy in patients visiting Korean medical institutions: Using electronic medical records (EMR) of 21 Korean medicine hospitals and clinics. J. Korean Med..

[4] Lee J.H., Woo H., Jang J.S., Kim J.I., Na Y.C., Kim K.R., Cho E., Lee J.H., Park T.Y. (2022). Comparison of concordance between Chuna manual therapy diagnostic methods (palpation, X-ray, artificial intelligence program) in lumbar spine: An exploratory, cross-sectional clinical study. Diagnostics.

[5] Lee J.S., Park K.W., Kim H.T., Park S.Y., Shin B.C. (2022). Systematic Review of the Diagnosis of Pelvic Deviation for Chuna Manual Therapy. J. Korean Med. Rehabil..

[6] Cibulka M.T., Koldehoff R. (1999). Clinical usefulness of a cluster of sacroiliac joint tests in patients with and without low back pain. J. Orthop. Sports Phys. Ther..

[7] Riddle D.L., Freburger J.K., North American Orthopaedic Rehabilitation Research Network (2002). Evaluation of the presence of sacroiliac joint region dysfunction using a combination of tests: A multicenter intertester reliability study. Phys. Ther..

[8] Cooperstein R., Hickey M. (2016). The reliability of palpating the posterior superior iliac spine: A systematic review. J. Can. Chiropr. Assoc..

[9] May S., Littlewood C., Bishop A. (2006). Reliability of procedures used in the physical examination of non-specific low back pain: A systematic review. Aust. J. Physiother..

[10] Hestbaek L., Leboeuf-Yde C. (2000). Are Chiropractic Tests for the Lumbo-Pelvic Spine Reliable and Valid? A Systematic Critical Literature Review. J. Manip. Physiol. Ther..

[11] Nolet P.S., Yu H., Côté P., Meyer A.L., Kristman V.L., Sutton D., Murnaghan K., Lemeunier N. (2021). Reliability and validity of manual palpation for the assessment of patients with low back pain: A systematic and critical review. Chiropr. Man. Ther..

[12] Kim T.G., Gi Y.M., Yang K.J., Lee K.B., Jo H., Choi J., Lee Y.J., Lee S., Ha I.H. (2018). The implications of X-ray use in Chuna manual therapy from the viewpoint of Korean medicine doctors. J. Acupunct. Res..

[13] Lee J.-H., Kim C.-G., Jo D.-C., Moon S.-J., Park T.-Y., Ko Y.-S., Nam H.-W., Lee J.-H. (2014). Diagnostic X-ray from the perspective of Chuna manual medicine, based on the listing system of spinal and pelvic subluxation. J. Korea CHUNA Man. Med. Spine Nerves.

[14] Lee J.M., Koog G.H., Choi B.M., Jeong H.A., Hong S.Y. (2010). The comparative study between leg length analysis and X-ray on diagnosis of pelvic malpositions: According to positions and valuation bases. J. Korea CHUNA Man. Med. Spine Nerves.

[15] Wall B.F., Kendall G.M., Edwards A.A., Bouffler S., Muirhead C.R., Meara J.R. (2006). What are the risks from medical X-rays and other low dose radiation?. Br. J. Radiol..

[16] Mehta B., Chockalingam N., Shannon T., Jevtic N., Lazic F., Jasani V., Eddison N., Healy A., Needham R. (2023). Non-invasive assessment of back surface topography: Technologies, techniques and clinical utility. Sensors.

[17] Diebo B.G., Shah N.V., Pivec R., Naziri Q., Patel A., Post N.H., Assi A., Godwin E.M., Lafage V., Schwab F.J. (2018). From static spinal alignment to dynamic body balance: Utilizing motion analysis in spinal deformity surgery. JBJS Rev..

[18] Domagalska-Szopa M., Szopa A. (2014). Gait pattern differences between children with mild scoliosis and children with unilateral cerebral palsy. PLoS ONE.

[19] Lee J.W., Ju M.S., Ko Y.S., Lim Y.G., Hwang H.H. (2023). Correlation Analysis about Subluxation Diagnosis Using Lumbar X-ray and Body Shape Analysis System. J. Korean Med. Rehabil..

[20] Kim K.-K., Haam J.-H., Kim B.T., Kim E.M., Park J.H., Rhee S.Y., Jeon E., Kang E., Nam G.E., Koo H.Y. (2023). Evaluation and Treatment of Obesity and Its Comorbidities: 2022 Update of Clinical Practice Guidelines for Obesity by the Korean Society for the Study of Obesity. J. Obes. Metab. Syndr..

[21] Levangie P.K. (1999). Four clinical tests of sacroiliac joint dysfunction: The association of test results with innominate torsion among patients with and without low back pain. Phys. Ther..

[22] Kim C.H., Kim J.Y., Shin B.C. (2006). A Study on the Pelvic Deviation According to Delivery Types, Frequency and Experience (A Radiological Study). J. Orient. Rehab. Med..

[23] Kurillo G., Hemingway E., Cheng M.L., Cheng L. (2022). Evaluating the accuracy of the Azure Kinect and Kinect V2. Sensors.

[24] Choi W., Kim H.T., Shin B.C., Lee S.H., Jang J.S. (2022). Reliability evaluation of 3D body surface measurement system for pelvic displacement diagnosis. JNCIST.

[25] Horsak B., Schwab C., Durstberger S., Thajer A., Greber-Platzer S., Kainz H., Jonkers I., Kranzl A. (2021). 3D free-hand ultrasound to register anatomical landmarks at the pelvis and localize the hip joint center in lean and obese individuals. Sci. Rep..

[26] Leardini A., Chiari L., Della Croce U., Cappozzo A. (2005). Human Movement Analysis Using Stereophotogrammetry. Part 3. Soft Tissue Artifact Assessment and Compensation. Gait Posture.

[27] Abbott J.H., Mercer S.R. (2003). Lumbar segmental hypomobility: Criterion-related validity of clinical examination items (a pilot study). N. Z. J. Physiother..

[28] Steyerberg E.W., Harrell F.E., Borsboom G.J., Eijkemans M.J., Vergouwe Y., Habbema J.D.F. (2001). Internal Validation of Predictive Models: Efficiency of Some Procedures for Logistic Regression Analysis. J. Clin. Epidemiol..

[29] Choi W., Jang J.S. (2026). A Fully Automated Deep Learning Pipeline for Anatomical Landmark Localization on Three-Dimensional Pelvic Surface Scans. Sensors.

