# Supplementary material for: Exploratory Agreement of 3D Body Surface Analysis with Expert Manual Assessment of Pelvic Malalignment in Patients with Non-Acute Low Back Pain: A Prospective Pilot Study with Linked Post-Hoc Analyses
Source: Diagnostics (Basel). 2026 Oct 10;16(20):3293. doi: 10.3390/diagnostics16203293 (PMC13703089; doi:10.3390/diagnostics16203293)
Supplement: Supplementary file 1 [file diagnostics-16-03293-s001.zip › 20260612_Supplementary material Table S1.pdf]

Supplementary Material

Supplementary Table S1. Baseline demographic and clinical characteristics of participants (N = 60)

Characteristic	Category	n (%)	Mean ± SD
**Sex**	Male	18 (30.0)	
	Female	42 (70.0)	
**Age, years**			37.97 ± 11.56
**NRS score a**	≥ 4 (inclusion criterion)	60 (100.0)	—
**LBP duration a**	> 2 weeks (inclusion criterion)	60 (100.0)	—
**BMI, kg/m²**			22.91 ± 3.08
**Height, cm**			164.85 ± 8.04
**Weight, kg**			62.48 ± 10.79
**Systolic BP, mmHg**			121.77 ± 13.86
**Diastolic BP, mmHg**			71.77 ± 10.89
**Pulse rate, beats/min**			83.73 ± 11.73
**Body temperature, °C**			36.45 ± 0.25
**Medical history**	Present	8 (13.3)	
	Absent	52 (86.7)	
**Medication history**	Present	8 (13.3)	
	Absent	52 (86.7)	
**Occupation**	Office worker	15 (25.0)	
	Professional	13 (21.7)	
	Self-employed	3 (5.0)	
	Service worker	1 (1.7)	
	Student	6 (10.0)	
	Homemaker	12 (20.0)	
	Unemployed	1 (1.7)	
	Other	9 (15.0)	

BMI, body mass index; BP, blood pressure; LBP, low back pain; NRS, numeric rating scale; SD, standard deviation.

a NRS score ≥ 4 and LBP duration > 2 weeks were inclusion criteria; continuous mean values were not collected separately.
